# Dynamic junction remodeling and transient vascular disruption during cancer cell extravasation across the blood-brain barrier

**DOI:** 10.3389/fcell.2026.1917794

**Published:** 2026-08-18

**Authors:** Yu Zhang, Shunze Deng, Yonggang Ren, Jiulin Du, Bing Xu

**Affiliations:** 1 School of Life Sciences, Nantong University, Nantong, Jiangsu, China; 2 Institute of Neuroscience, Center for Excellence in Brain Science and Intelligence Technology, Chinese Academy of Sciences, Shanghai, China

**Keywords:** brain metastasis, extravasation, knockin, VE-cadherin, zebrafish

## Abstract

Brain metastasis is the major cause of cancer-related mortality and remains a critical challenge in clinical oncology. Extravasation of circulating cancer cells across blood-brain barrier (BBB) is the first crucial and rate-limiting step for brain metastasis growth. However, the dynamic changes of brain endothelial cell junction and vascular integrity during cancer cell extravasation across BBB remains unclear. Here, we established a knockin zebrafish line, *Ki(cdh5-eGFP)*, in which EGFP is fused to the C-terminal of endogenous VE-cadherin, enabling *in vivo* real-time imaging of brain endothelial junction dynamics. Then, we established a brain metastasis model in *Ki(cdh5-eGFP)* zebrafish, and tracked the multistep cascade of cancer cell extravasation across BBB. We demonstrated that cancer cells cross brain vascular endothelial cells, accompanied by dynamic remodeling of VE-cadherin and transient disruption of vascular integrity. Our model transforms the BBB into a dynamically observable interface, enabling real-time visualization of junction remodeling during cancer cell extravasation, which will not only facilitate further mechanistic study, but also provide a valuable platform for preclinical drug screening.

## Introduction

Brain metastases are the most common malignant tumors in the central nervous system, with limited treatment options and extremely poor prognosis (Achrol et al., 2019; Aizer et al., 2022). Metastatic dissemination involves multiple steps, including local invasion of the primary tumor, intravasation, survival in transit, arrest, extravasation and colonization (Achrol et al., 2019; Dai et al., 2025; Strilic and Offermanns, 2017). Among these, cancer cell extravasation from brain microcapillaries is the first crucial and rate-limiting step for the seeding of brain metastases (Achrol et al., 2019). So far, the underlying cellular and molecular processes governing cancer cell extravasation from the blood-brain barrier (BBB) remain largely unknown.

Evidence has been provided that cancer cell extravasation resembles leukocyte transendothelial migration, involving the disruption of endothelial cell (EC) junctions and subsequent transmigration across the endothelial layer (Dupas et al., 2024). However, almost all the evidence comes from *in vitro* models or endpoint *in vivo* experiments (Strilic and Offermanns, 2017). Recent studies using mouse brain metastasis model and intravital two-photon imaging showed active EC remodeling, including inducing thin luminal EC projections and vascular looping, during and after cancer cell extravasation *in vivo* (Kienast et al., 2010; Karreman et al., 2023). However, there is still a lack of direct *in vivo* evidence on whether and how cancer cells alter EC junction and BBB integrity during extravasation.

Zebrafish is a powerful vertebrate model for *in vivo* visualization of vascular and cancer cell dynamics due to its optical transparency and genetic trackability (Bahrami and Childs, 2020; Patton et al., 2021; Osmani and Goetz, 2019). Multiple zebrafish xenograft models have been established to investigate cancer metastasis (Astell and Sieger, 2020; Letrado et al., 2018). Most of these models focus on the trunk region (Peralta et al., 2025; Asokan et al., 2020; Osmani et al., 2019), and brain metastasis studies have largely relied on direct intracranial injection of cancer cells - an approach that bypasses the early steps of metastatic dissemination (Almstedt et al., 2022; Ai et al., 2022; Fan et al., 2021). Consequently, the application of zebrafish to study brain metastasis, particularly the dynamic remodeling of brain endothelial junctions during cancer cell extravasation, remains limited. A major obstacle is the lack of zebrafish models that faithfully label endogenous endothelial junction proteins, thereby hindering direct visualization of junctional dynamics *in vivo*.

The junctional components of BBB primarily consist of tight and adherens junctions, which work synergistically to maintain BBB integrity (Profaci et al., 2020). Vascular endothelial cadherin (VE-cadherin) is the core transmembrane protein of adherens junctions, which not only maintains vascular integrity, but also directly influences vascular permeability (Daneman and Prat, 2015; Xu et al., 2017; Giannotta et al., 2013). Real-time visualization of endogenous VE-cadherin *in vivo* is essential to unravel how endothelial junctions respond to cancer cell extravasation. Current approaches for labeling proteins in zebrafish typically rely on ectopic expression driven by transgenic promoters or bacterial artificial chromosome (BAC) constructs (Kwan et al., 2007; Fuentes et al., 2016). For instance, *Tg(UAS:VE-cadherinΔC-EGFP)* has been used to study the dynamics of endothelial junction during angiogenesis and pruning (Lenard et al., 2013; Lenard et al., 2015), and *TgBAC(ve-cad:ve-cadTS)* has been applied to analyze junctional tension and VE-cadherin distribution (Lagendijk et al., 2017). While useful, these models do not faithfully recapitulate the endogenous expression and dynamics of VE-cadherin during vascular development. Thus, direct knock-in tagging at the endogenous locus will provide the most accurate approach to study the VE-cadherin dynamics under physiological and pathological conditions, but this has been technically challenging in zebrafish until recently (Auer et al., 2014; Li et al., 2015). To date, the change of VE-cadherin during cancer cell extravasation in brain is still unclear.

Here, to optimally observe the cellular process of cancer cell extravasation and the dynamic change of VE-cadherin in brain, we generated a zebrafish line labeling endogenous VE-cadherin and established a brain metastasis model in this system to directly track the dynamic cellular and molecular processes during cancer cell extravasation, at the whole-brain and single-cell levels. We observed that cancer cells extravasate across BBB accompanied by redistribution and transient local reduction of VE-cadherin, BBB disruption, and subsequent junction resealing. Our animal model not only facilitates further mechanistic studies, but also provides a valuable platform for preclinical drug screening.

## Results

### Establishment of *Ki(cdh5-eGFP)* zebrafish line

**FIGURE 1:**
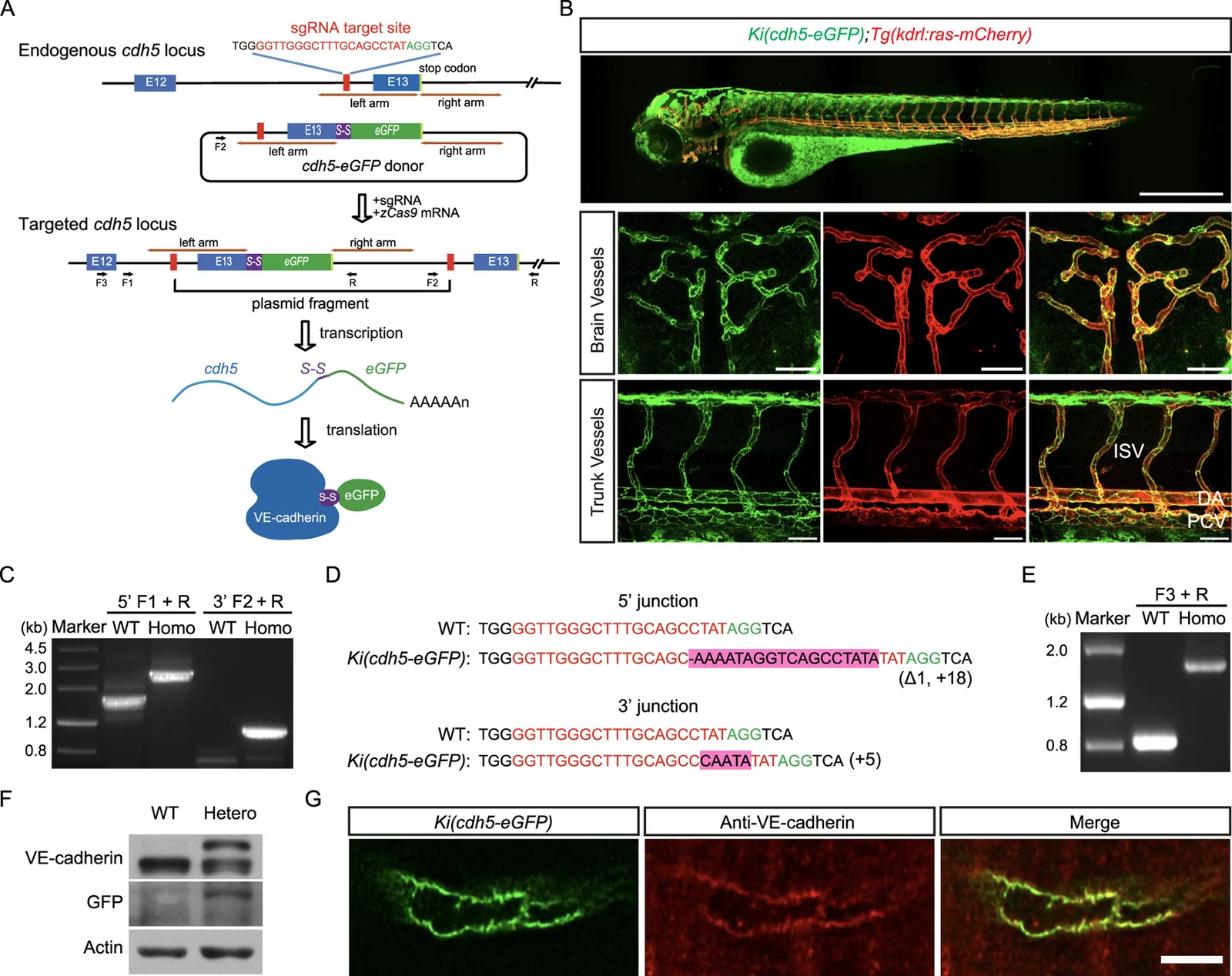
Establishment of *Ki(cdh5-eGFP)* zebrafish. **(A)** Schematic of CRISPR/Cas9-mediated knockin strategy. The *cdh5-eGFP* donor plasmid was co-injected with sgRNA and zCas9 mRNA at the one-cell stage. Cas9-mediated cleavage of the genomic locus and donor plasmid enabled insertion of *cdh5-eGFP* into the last intron of *cdh5*, generating an eGFP-tagged VE-cadherin protein. S–S: serine-serine linker; Red font: sgRNA target site; green font: PAM. **(B)** Representative image of a 3 dpf *Ki(cdh5-eGFP);Tg(kdrl:ras-mCherry)* larva showing endothelial junction labeling in brain and trunk vessels. The green signals on the skin and yolk sac are autofluorescence. ISV, intersegmental vessel; DA, dorsal aorta; PCV, posterior cardinal vein. Scale bars: top, 500 μm; bottom, 50 μm. **(C)** PCR verification of knock-in alleles. Primers F1/R yielded a 1756 bp band in WT and a 2476 bp band in homozygous knock-in fish. Primers F2/R amplified a 1095 bp band only in homozygous knock-ins. **(D)** Sequencing of 5′and 3′junctions showing minor indels (highlighted in magenta). Red font: sgRNA target site; green font: PAM. **(E)** RT-PCR showing an 868 bp band in WT and a 1600 bp band in homozygous *Ki(cdh5-eGFP)*. **(F)** Western blot of wild-type and heterozygous larvae. Anti-VE-cadherin detected both WT (∼120 kDa) and VE-cadherin-eGFP (∼150 kDa). Anti-GFP detected only the fusion protein. **(G)** Immunofluorescence of *Ki(cdh5-eGFP)* larvae showing colocalization of VE-cadherin staining (red) with eGFP fluorescence (green) in the trunk vessel. Scale bar, 25 μm.

To directly visualize the dynamic changes of endogenous VE-cadherin *in vivo*, we generated a knockin zebrafish line, *Ki(cdh5-eGFP)*, in which EGFP is fused to the C-terminal of endogenous VE-cadherin by CRISPR/Cas9-mediated knockin. Briefly, a single-guide RNA (sgRNA) targeting the last intron of *cdh5*, together with a donor plasmid carrying the *cdh5-eGFP* cassette and *zCas9* mRNA, were injected into one-cell-stage embryos to enable non-homologous end joining-mediated knockin (Figure 1A, ref (Li et al., 2015)). After fluorescence screening, we identified one F0 founder (1/20). To examine whether the *Ki(cdh5-eGFP)* specifically labels all vascular junctions, we crossed it with the *Tg(kdrl:ras-mCherry)* line, in which vascular endothelial cell membranes are labeled with mCherry. The *Ki(cdh5-eGFP);Tg(kdrl:ras-mCherry)* larva exhibit robust endothelial junction-specific EGFP expression throughout the vasculature, including both brain and trunk vessels (Figure 1B). We further verified the correct genomic insertion of *eGFP* with PCR using three primer pairs targeting both donor and genomic regions (Figure 1A). PCR products of the expected sizes were detected in homozygous *Ki(cdh5-eGFP)* embryos (Figure 1C). Junction sequencing revealed minor indels at both 5′and 3′ends (Figure 1D). As the insertion occurred within an intron, these indels did not interfere with *cdh5* expression. Reverse transcription PCR confirmed expression of the *cdh5-eGFP* transcript, producing a band ∼800 bp longer than that from wild-type (WT) fish (Figure 1E).

Western blot analysis further validated correct protein expression. In heterozygous *Ki(cdh5-eGFP)* larvae, two bands were detected with an anti-VE-cadherin antibody: the WT protein (∼120 kDa) and the VE-cadherin-eGFP fusion protein (∼150 kDa), while an anti-eGFP antibody detected only the fusion protein (∼150 kDa) (Figure 1F). Notably, the expression levels of WT and fusion proteins in heterozygotes were nearly equivalent, indicating that knock-in tagging did not disrupt endogenous VE-cadherin expression. Immunofluorescence staining confirmed that eGFP signals were well colocalized with VE-cadherin immunolabeling in the trunk vessels (Figure 1G). Collectively, these results demonstrated that *Ki(cdh5-eGFP)* effectively labels endogenous VE-cadherin protein.

### VE-cadherin dynamics during angiogenesis and vessel pruning

**FIGURE 2:**
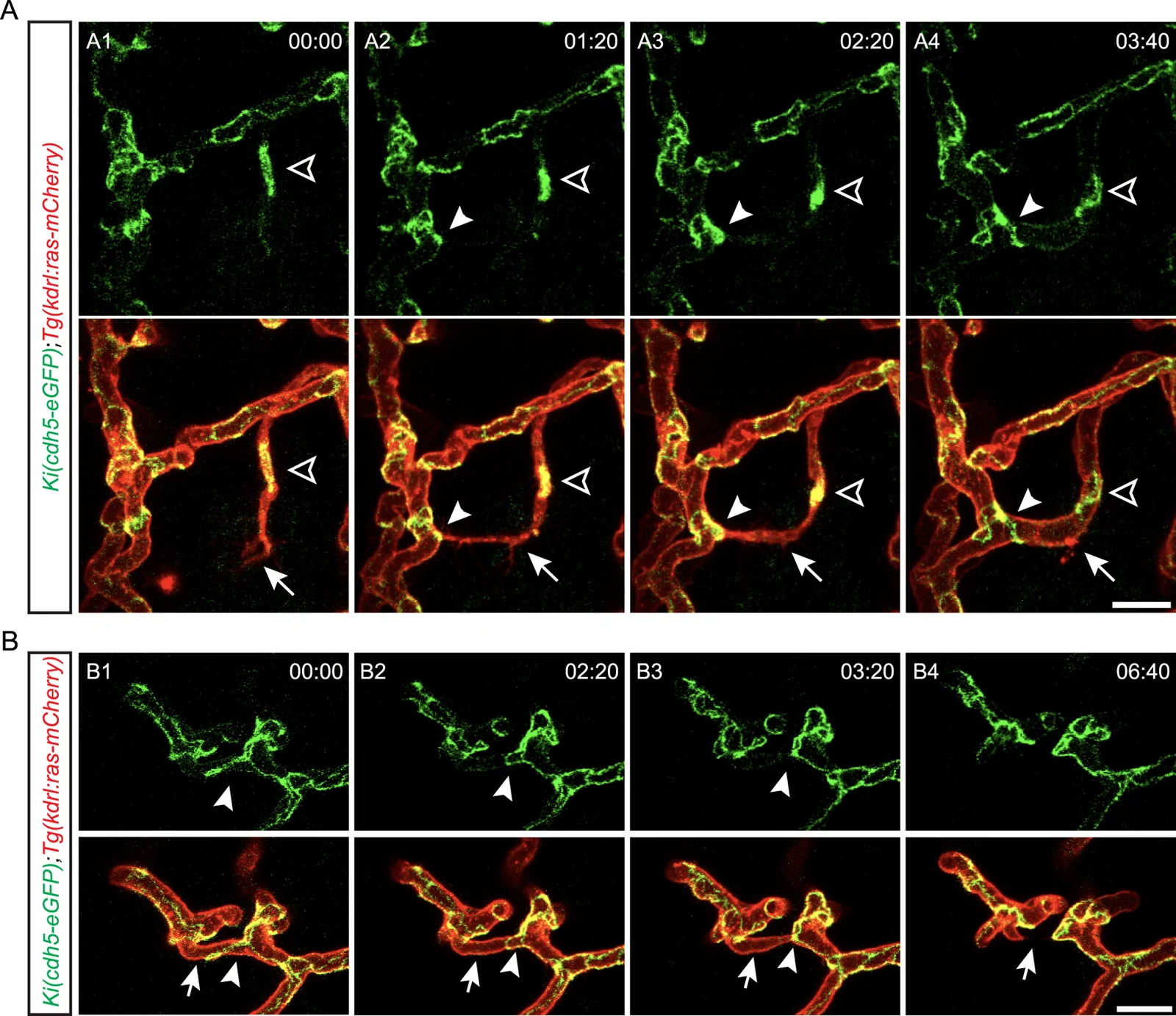
VE-cadherin dynamics during angiogenesis and vessel pruning. **(A)** Time-lapse imaging of sprouting angiogenesis in a 60 hpf *Ki(cdh5-eGFP);Tg(kdrl:ras-mCherry)* larva. Tip cell (ETC) filopodia (arrow) show low VE-cadherin expression during exploration (A1). VE-cadherin accumulates between ETC and stalk cell (empty arrowhead). Upon contact with a target vessel, a VE-cadherin spot forms (arrowhead, A2), reorganizes into a ring (A3), and expands during lumen formation (A4). Time stamps: hh:mm. Scale bar, 20 μm. **(B)** Time-lapse imaging of vessel pruning in a 60 hpf *Ki(cdh5-eGFP);Tg(kdrl:ras-mCherry)* larva. VE-cadherin rings at the junction between pruning (arrow) and adjacent vessels retract (B1–B2), leaving a discrete VE-cadherin spot (arrowhead, B3). The pruning vessel disconnects and retracts into the adjacent vessel (B4). Time stamps: hh:mm. Scale bar, 20 μm.

Next, to assess whether *Ki(cdh5-eGFP)* accurately indicates the dynamic changes of endogenous VE-cadherin during EC junction remodeling, we tracked the VE-cadherin-EGFP signals during sprouting angiogenesis and vessel pruning in the brain. We performed *in vivo* time-lapse imaging of 2.5 days post-fertilization (dpf) *Ki(cdh5-eGFP);Tg(kdrl:ras-mCherry)* larvae, in which vascular endothelial cell membranes are labeled with mCherry. We observed characteristic VE-cadherin changes during sprouting angiogenesis (Figure 2A, Supplementary Video S1), including formation of VE-cadherin puncta upon initial contact between endothelial tip cells (ETCs) and target vessels, expansion into ring-like structures during lumen formation, and dynamic reorganization between ETCs and stalk cells during vascular maturation. Different from previous study using transgenic zebrafish *Tg(uas:cdh5ΔC-eGFP)* (Lenard et al., 2013; Sauteur et al., 2014), in which the cytoplasmic domain of exogenous VE-cadherin was replaced by EGFP, we found endogenous VE-cadherin signaling was largely absent from ETC filopodia (Figures 2A–A1). Nonetheless, we cannot exclude the possibility that the VE-cadherin expression may be too low to be detected in *Ki(cdh5-eGFP)*. During vessel pruning, we observed the ring-like structure formed by VE-cadherin underwent progressive constriction, and ultimately converging to a single point (Figures 2B-B1–B3; Supplementary Video S2), following by endothelial cell disconnection and the fusion of regressing vessel into adjacent vessel (Figures 2B–B4). Together, these observations confirmed that *Ki(cdh5-eGFP)* accurately captures the endogenous VE-cadherin dynamics during EC junction remodeling.

### Transient disruption of blood vessels during cancer cell extravasation

**FIGURE 3:**
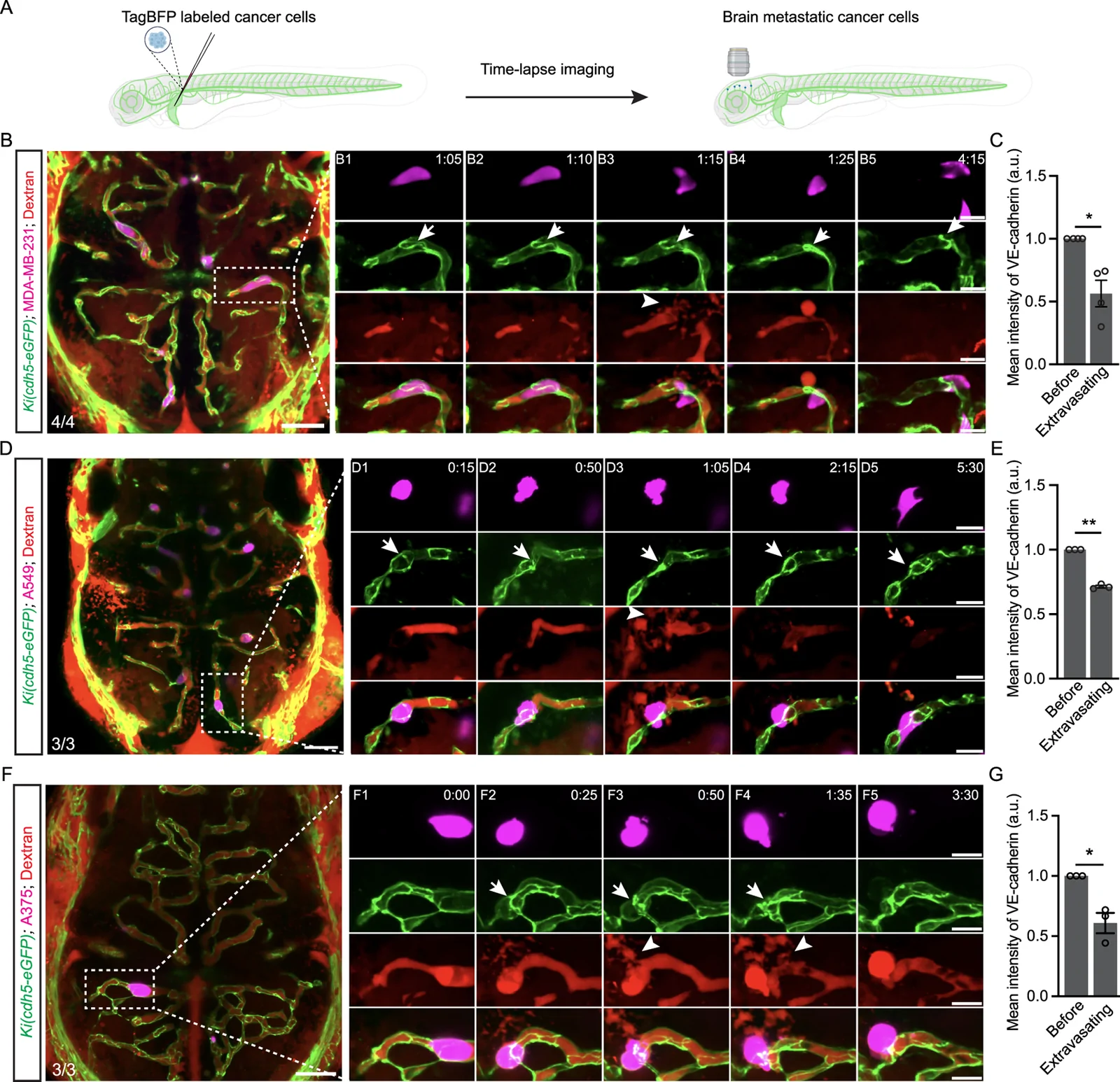
Transient brain vascular disruption during cancer cell extravasation. **(A)** Schematic of zebrafish brain metastatic model. TagBFP-labeled cancer cells were injected into the common cardinal vein of 3 dpf *Ki(cdh5-eGFP)* larvae. Time-lapse imaging was used to track extravasation events in brain vessels. **(B)** Brain metastasis of MDA-MB-231 cells. Cell arrest within a vessel (B1), local decrease in VE-cadherin at the junction (arrows) (B2), extravasation through endothelial junctions with dextran leakage (arrowhead) (B3), VE-cadherin condensation at the disrupted junction (B4), junctional reorganization restores vessel integrity (B5). Time stamps: hh:mm. Scale bars: left, 50 μm; right, 20 μm. **(C)** Quantification of VE-cadherin intensity during MDA-MB-231 cell brain metastasis. Four brain metastatic events were analyzed. *P* = 0.0256. **(D)** Brain metastasis of A549 cells.Cell arrest (D1), VE-cadherin downregulation (D2), dextran leakage and VE-cadherin condensation (D3), vascular repair (D4), and abluminal attachment of the extravasated cell (D5). Time stamps: hh:mm. Scale bars: left, 50 μm; right, 20 μm. **(E)** Quantification of VE-cadherin intensity during A549 cell brain metastasis. Three brain metastatic events were analyzed. *P* = 0.0013. **(F)** Brain metastasis of A375 cells: Cell arrest (F1 & F2), VE-cadherin downregulation and dextran leakage (F3), dextran leakage and VE-cadherin condensation (F4), vascular repair (F5). Time stamps: hh:mm. Scale bars: left, 50 μm; right, 20 μm. **(G)** Quantification of VE-cadherin intensity during A375 cell brain metastasis. Three brain metastatic events were analyzed. *P* = 0.0438. The arrows indicate the VE-cadherin expression and distribution; the arrowheads indicate the leakage of dextran accompanied with the extravasation of cancer cells. Data presented as mean ± SEM. Statistical significance determined by One sample *t* and Wilcoxon test.

Then, to observe the cellular and molecular processes of cancer cell extravasation across BBB, we established a brain metastasis model in *Ki(cdh5-eGFP)* zebrafish. Briefly, TagBFP-labeled cancer cells were co-injected with Alexa 568-dextran into the circulation of 3-dpf *Ki(cdh5-eGFP)* larvae via the common cardinal vein, and then time-lapse confocal imaging was conducted to track the process of cancer cell extravasation (Figure 3A, Supplementary Video S3). Furthermore, only a small fraction of injected cancer cells (<10%) successfully extravasated into the brain, allowing us to trace the extravasation process at the single-cell level. Given that brain metastases most commonly originate from lung cancer, breast cancer and melanoma clinically (Fecci et al., 2019), we therefore examined the extravasation process in these three cancer cell types. We first examined the triple-negative breast cancer cells, MDA-MB-231, which is widely used in the studies of brain metastases. We observed the multistep cascades during cancer cell extravasation across BBB (Supplementary Video S4). Initially, MDA-MB-231 cancer cells arrest in the brain microvasculature, transiently tether to the inner wall of blood vessels (Figures 3B–B1). Subsequently, cancer cells firmly adhere to brain endothelial cells and lead to the change of VE-cadherin distribution, with significant VE-cadherin signal decrease in the local area where tumor cells were about to extravasate (Figures 3B–B2, 3C), while the VE-cadherin intensity in the opposite normal vessels was not changed (Supplementary Figure S1A). Finally, MDA-MB-231 cancer cells change shape and cross the endothelial barrier, accompanied by persistent VE-cadherin disruption and dextran leakage, indicating disruption of vascular integrity (Figure 3B). Notably, this disruption of vascular integrity is transient, as VE-cadherin reassembles first into patchy clusters, then redistribute into a ring-like structure and ultimately restore continuous linear junctions after cancer cells have fully extravasated, leading to vascular integrity repair and vessel recanalization, as evidenced by the flow of cancer cell through the repaired vessel (Figures 3B-B4–B5, Supplementary Video S4). The extravasated MDA-MB-231 cancer cells are then localized along the abluminal vessel surface and adapt a migratory morphology conducive for further dissemination (Figures 3B–B5).

To test if the transient brain vascular disruption during extravasation is a common phenotype during brain metastasis, we further investigated the extravasation process of A549 lung cancer cells (Figures 3D,E; Supplementary Video S5) and A375 melanoma cells (Figures 3F,G; Supplementary Video S6) under the same experimental conditions. We found the same dynamic extravasating processes in both cell lines, including persistent VE-cadherin downregulation during tumor cell extravasation, without VE-cadherin change in the opposite normal vessels (Supplementary Figures S1B,C), transient vascular integrity disruption, patchy-to-ring-like reassembly of VE-cadherin and vascular repair after tumor cell extravasation. These results demonstrate that the extravasation, with dynamic remodeling of junctional protein VE-cadherin and transient disruption of vascular integrity, is a general phenomenon shared across multiple tumor cell lines during brain metastasis.

## Discussion

In summary, taking advantage of the newly generated *Ki(cdh5-eGFP)* zebrafish line and the brain metastasis model built upon it, we for the first time provide direct *in vivo* evidence that cancer cells extravasate across brain vascular endothelial cells, accompanied by dynamic remodeling of VE-cadherin and transient disruption of vascular integrity.

It is worth noting that our findings differ from previous observations in trunk blood vessels. In trunk vessels, tumor cells were reported to extravasate by engulfment of endothelial cells, and this process was not accompanied by detectable vascular leakage (Follain et al., 2018; Stoletov et al., 2010; Stoletov et al., 2007). In contrast, we demonstrate that cancer cells cross brain vascular endothelial cells via the paracellular route, which involves dynamic remodeling of the junctional protein VE-cadherin and transient disruption of vascular integrity, followed by junctional resealing, vascular integrity repair and vessel recanalization after extravasation. The distinct mechanisms observed in the brain versus trunk vasculature suggest a brain-specific mode of endothelial junction remodeling that governs tumor cell extravasation across brain vasculature.

Recent studies have revealed the molecules involved in cancer cell transendothelial migration and brain metastatic outgrowth (Fecci et al., 2019; Reymond et al., 2013). For instance, cathepsin S expression in cancer cells proteolyses junctional adhesion molecule-B (JAM-B) to facilitate extravasation cross BBB (Sevenich et al., 2014), ADAM8 expression in breast cancer cells promotes β1-integrin-dependent transendothelial migration (Romagnoli et al., 2014), and ST6GALNAC5 expression in breast cancer cells enhances transendothelial migration and extravasation cross BBB (Bos et al., 2009). Meanwhile, enhancement or preservation of BBB integrity decreases brain metastatic growth (Fecci et al., 2019). In this study, we demonstrated that adherens junction protein VE-cadherin is dynamically changed during cancer cell extravasation across the BBB, indicating VE-cadherin not only provides an essential mechanical adhesive force to maintain vascular integrity, but may also influences vascular permeability and cancer cell extravasation through its dynamic regulation. Further research is needed to clarify whether and how cancer cells regulate VE-cadherin dynamics to facilitate extravasation.

As the dynamic process of brain metastasis cannot be faithfully recapitulated by *in vitro* or endpoint *in vivo* models, the *in vivo* time-lapse imaging of *Ki(cdh5-eGFP)* zebrafish offers a powerful platform for future investigations of this process. This approach transforms the BBB into a dynamically observable interface, enabling real-time visualization of how cancer cells adhere to ECs, open endothelial junctions, and invade the brain parenchyma. Future studies can leverage this toolkit to direct visualize BBB disruption, dissect molecular mechanisms of cancer cell-induced junctional disassembly upon EC contact, and validate drugs that disrupt cancer cell-EC interactions. Ultimately, this live-imaging approach will not only advance mechanistic understanding of brain metastasis, but also accelerate clinical translation of BBB-protective and anti-metastatic strategies.

## Materials and methods

### Zebrafish husbandry

Adult zebrafish were maintained in an automated housing system (Haisheng, Shanghai, China) under a 14:10 h light–dark cycle. Embryos and larvae were raised in egg water (60 mg Instant Ocean Sea Salts per 1 L water) at 28 °C under the same light–dark cycle. The *Tg(kdrl:ras-mCherry)* line was used to fluorescently label endothelial cells. To suppress pigmentation, embryos intended for live imaging beyond 24 h post-fertilization (hpf) were treated with 0.004% phenylthiourea (PTU). All procedures were performed in accordance with the animal care guidelines of Nantong University.

### Generation of the *Ki(cdh5-eGFP)* zebrafish line

The *Ki(cdh5-eGFP)* line was generated using CRISPR/Cas9-mediated knock-in as previously described (Li et al., 2015). A gRNA target site (GGT​TGG​GCT​TTG​CAG​CCT​AT) located in the intron between the penultimate and final exon of *cdh5* was selected for donor insertion. Oligodeoxynucleotides with BbsI overhangs were annealed and ligated into the BbsI-linearized pT7-gRNA vector. PCR with primers T7-cr F and Tracr R yielded a 130 bp DNA fragment containing the T7 promoter and gRNA sequence, which was purified (QIAquick Gel Extraction Kit, QIAGEN) and used for *in vitro* transcription (MAXIscript T7 Kit, Ambion). zCas9 mRNA was transcribed from pGH-T7-zCas9 using the mMESSAGE mMACHINE T7 Ultra Kit (Ambion).

The donor construct consisted of a left homology arm containing the gRNA target site, the *eGFP* sequence, and a right homology arm, synthesized and cloned into the PM19-T vector. A 1 nL injection mix containing 800 pg zCas9 mRNA, 80–100 pg gRNA, and 15 pg donor plasmid was delivered into one-cell stage embryos. At 3 dpf, 10 injected embryos were screened for knock-in efficiency by PCR with primers F1/R1, and the remaining embryos were raised to adulthood. Potential founders were outcrossed with WT fish, and F1 progeny were screened by PCR (F1/R, F2/R). Stable F2 lines were established for subsequent experiments.

Primers used:

T7-cr F: 5′-GAA​ATT​AAT​ACG​ACT​CAC​TAT-3′

Tracr R: 5′-AAA​AAA​AGC​ACC​GAC​TCG​GTG​CCA​C-3′

F1: 5′-TGG​GGC​AAT​ATT​CCA​ACT​TCC​A-3′

F2: 5′-TGT​AAA​ACG​ACG​GCC​AGT-3′

R: 5′-CAT​TTT​GTC​AAT​CAC​CAG​GTG​GTC-3′

### Reverse transcription PCR

Total RNA was extracted from 3 dpf WT or *Ki(cdh5-eGFP)* larvae using the FastPure® Cell/Tissue Total RNA Isolation Kit V2 (Vazyme). cDNA was synthesized from 500 ng RNA with HiScript® III All-in-one RT SuperMix (Vazyme). PCR amplification using primers F3/R confirmed expression of the *cdh5-eGFP* fusion transcript.

Primers:

F3: 5′-CGC​CAT​CCT​TCT​CTG​TAT​CAT​TAC​C-3′

R: 5′-CAT​TTT​GTC​AAT​CAC​CAG​GTG​GTC-3′

### Cell culture

Breast cancer cells (MDA-MB-231) were provided by Liming Chen (Nanjing Normal University), and lung cancer cells (A549) were purchased from Procell (CL-0016). MDA-MB-231 cells were cultured in DMEM (Gibco) supplemented with 10% FBS (CELLiGENT) and 1% penicillin/streptomycin (Gibco). A549 cells were cultured in RPMI-1640 (Gibco) with 10% FBS and 1% penicillin/streptomycin. Cells were dissociated using TrypLE (Gibco) and transduced with a lentiviral vector carrying TagBFP under the CMV promoter. After 48 h, TagBFP-positive cells were isolated by flow cytometry and expanded for experiments.

### Transplantation of cancer cell in zebrafish

At 3 dpf, larvae were anesthetized with 0.2 mg/mL tricaine (Sigma-Aldrich) and embedded in 1% low - melting agarose (Invitrogen). TagBFP-MDA-MB-231, TagBFP-A549, or TagBFP-A375 cells were dissociated, resuspended in HBSS at 2 × 10^7^ cells/mL, and mixed with 1 mg/mL Alexa568 - dextran (Invitrogen). Approximately 200 tumor cells were injected into the common cardinal vein (CCV) of each larva. Successful injections were confirmed by the presence of TagBFP-positive cells in the posterior cardinal vein, and larvae were selected for time-lapse confocal imaging.

### *In vivo* confocal imaging

Imaging was performed on a Nikon NiE-A1 confocal microscope with a ×20 water-immersion objective (NA = 1.15). Larvae were embedded in 1% low-melting agarose on glass-bottom dishes. Z-stacks were acquired at 1 μm intervals. Time-lapse intervals were set to 20 min for angiogenesis/pruning studies and 5 min for cancer metastasis assays. Images were processed using Fiji (NIH) (Schindelin et al., 2012).

### Quantification of the VE-cadherin intensity

The time-lapse videos were carefully analyzed to identify the time point and extravasation site of cancer cells. We then used the “Rectangle” selection tool in Fiji (NIH) to outline the extravasation site. VE-cadherin intensity within this selected region was measured at two time points—before extravasation and during extravasation—using the “Measure” function. The mean intensities at these two time points were then compared and analyzed.

### Western blot

At 3 dpf, larvae were lysed in 200 μL RIPA buffer (Beyotime) containing protease inhibitors (Merck). Lysates were cleared by centrifugation (13,000 g, 15 min, 4 °C). Equal amounts of protein were separated by SDS-PAGE and transferred to PVDF membranes. After blocking (TBS +0.2% Triton X-100 + 5% BSA, 2 h), membranes were incubated overnight with primary antibodies, washed, and incubated with HRP-conjugated secondary antibodies (Jackson). Bands were visualized with ECL substrate (Pierce). Primary antibodies: VE-cadherin (LSBio, LS-C210049); GFP (Invitrogen, LOT:1,661,233); Actin (Abmart, M20010).

### Immunofluorescence

Larvae (3 dpf) were fixed overnight in 4% PFA in PBST (0.1% Triton X-100) at 4 °C. After washing, they were permeabilized in cold acetone (−20 °C, 10 min) and blocked in PBST containing 5% goat serum for 1 h. Samples were incubated with anti-VE-cadherin antibody (LSBio, LS-C210049, 1:200) overnight at 4 °C, washed, and then incubated with Alexa546 goat anti-rat secondary antibody (ThermoFisher, A-11081, 1:500) overnight. After washing, larvae were imaged directly.

### Statistical analysis

All statistical analyses were performed using GraphPad Prism (version 9.5). One sample t and Wilcoxon test was used. Data were shown as ± SEM. n.s no significance, *P < 0.05, **P < 0.01. The exact *P* values were indicated in figure legends.

## Data Availability

The original contributions presented in the study are included in the article/Supplementary Material, further inquiries can be directed to the corresponding authors.

## Supplementary material

SUPPLEMENTARY MATERIAL VIDEO S1 Sprouting angiogenesis in a 60 hpf *Ki(cdh5-eGFP);Tg(kdrl:ras-mCherry)* larva. An endothelial tip cell extends toward and connects with a target vessel. Interval: 20 min. Scale bar, 20 μm.

SUPPLEMENTARY MATERIAL VIDEO S2 Vessel pruning in a 60 hpf *Ki(cdh5-eGFP);Tg(kdrl:ras-mCherry)* larva. Retraction and fusion of regressing vessels are visualized by VE-cadherin dynamics. Interval: 20 min. Scale bar, 20 μm.

SUPPLEMENTARY MATERIAL VIDEO S3 Brain metastasis assay in a 3 dpf *Ki(cdh5-eGFP)* larva injected with TagBFP-MDA-MB-231 cells (magenta) and dextran. Extravasation site indicated by a white arrow. Interval: 5 min. Scale bar, 50 μm.

SUPPLEMENTARY MATERIAL VIDEO S4 Enlarged time-lapse of extravasation in a 3 dpf *Ki(cdh5-eGFP)* larva injected with TagBFP-MDA-MB-231 cells. Interval: 5 min. Scale bar, 20 μm.

SUPPLEMENTARY MATERIAL VIDEO S5 Brain metastasis assay in a 3 dpf *Ki(cdh5-eGFP)* larva injected with TagBFP-A549 cells (magenta) and dextran. Interval: 5 min. Scale bar, 20 μm.

SUPPLEMENTARY MATERIAL VIDEO S6 Brain metastasis assay in a 3 dpf *Ki(cdh5-eGFP)* larva injected with TagBFP-A375 cells (magenta) and dextran. Interval: 5 min. Scale bar, 20 μm.
