# Supplementary material for: Dynamic junction remodeling and transient vascular disruption during cancer cell extravasation across the blood-brain barrier
Source: Front Cell Dev Biol. 2026 Aug 18;14:1917794. doi: 10.3389/fcell.2026.1917794 (PMC13529746; doi:10.3389/fcell.2026.1917794)
Supplement: Supplementary file 1 [file DataSheet1.docx]

**Supplementary Figure 1.** Quantification of VE-cadherin intensity in the contralateral site of cancer cell extravasation during MDA-MB-231 (A), A549 (B), and A375 (C) brain extravasation. Data presented as mean ± SEM. Statistical significance determined by One sample *t* and Wilcoxon test.
